# 

**Published:** 1812-09

**Authors:** 


### MONTHLY CATALOGUE OF MEDICAL BOOKS.

PRACTICAL Observations on Etropium, or Eversion of the Eye-lids, with a Description of a new Operation for the Cure of that Disease; on the Modes of forming an Artificial Pupil; and on Cataract. Illustrated with colored Plates. By William Adams, Member of the Royal College of Surgeons, London, &c. 8vo.—Callow.

A Letter on the State and Condition of Apothecaries; with Proposals for making their offices more respectable and more beneficial to the Public. Addressed to Pharmacopola Verus, by a True Surgeon. 8vo.—Callow.

Medical Observations and Inquiries. By a Society of Physicians in London. The Second Edition of Vol. VI. 8vo.—Callow.

A Treatise on Veterinary Medicine, in Three Volumes—Vol. III, containing practical Observations on some important Diseases of the Horse; viz. the Glanders, Farcy, Staggers, Inflammation of the Lungs and Bowels, the prevention and treatment of Lameness, and Precautions to be observed in Purchasing Horses. By James White. 12mo.—Longman and Co.

---

### *NOTICES TO CORRESPONDENTS.*

*Mr. Stevenson's Paper on Cataract will be inserted as soon as the Engraving is completed.*

*Communications have been received from the following Gentlemen, and will appear in due course:—From D. Thompson, M.D.; Robert Kinglake, M.D.; Messrs. R. W. Bamfield, R. Harrup, R. Charnock, J. Forster; an impartial Medical Man; H.; X. Y. Z.; &c. &c.*

*We are requested by Dr. Pigot to inform the Public, that "the calculations of Medicus, respecting the quantity of medicine given in Dr. Pigot's case of fever, must be erroneous, his premises being incorrect."*

